# A Brief Review on the Role of the Transcription Factor PBX1 in Hematologic Malignancies

**DOI:** 10.3390/ijms262110545

**Published:** 2025-10-30

**Authors:** Sofia Chatzileontiadou, Kassiani Boulogeorgou, Christina Frouzaki, Maria Papaioannou, Triantafyllia Koletsa, Evdoxia Hatjiharissi

**Affiliations:** 1 Division of Hematology, 1st Department of Internal Medicine, AHEPA University Hospital, Aristotle University of Thessaloniki, 54636 Thessaloniki, Greece; schatzil@auth.gr (S.C.); cfrouza@auth.gr (C.F.); papaioam@auth.gr (M.P.); 2 Department of Pathology, School of Medicine, Aristotle University of Thessaloniki, University Campus, 54124 Thessaloniki, Greece; kboulog@auth.gr

**Keywords:** PBX1, E2A::PBX1, hematologic neoplasms, multiple myeloma, lymphoma, hematopoiesis

## Abstract

Pre-B-cell leukemia factor 1 (PBX1) is a transcription factor that plays a significant role in various physiological, developmental, and oncogenic processes in humans. The mechanisms and interactions of PBX1 in both solid and hematologic malignancies remain significant areas of study. It was initially found in pre-B-cell acute lymphoblastic leukemia as a result of the chromosomal translocation t(1;19). Over the years, its role in other blood neoplasms has been studied. PBX1 and its variant E2A::PBX1 regulate gene expression that influences cell proliferation and differentiation in hematopoietic lineages. Their interaction with oncogenic partners results in abnormal gene regulation and tumorigenesis. Research has predominantly focused on the role of these factors in leukemias and plasma cell neoplasms, whereas other hematologic neoplasms have been largely overlooked. The potential application of PBX1 as a prognostic and predictive biomarker has recently gained attention. However, further research is needed to fully understand its complex role and how it can be targeted for therapeutic purposes. This review summarizes current knowledge on PBX1’s role in the growth of both mature and immature hematologic neoplasms. Moreover, it focuses on its prospective use as a therapeutic target and to predict prognosis, especially for aggressive neoplasms that do not respond to current therapeutic approaches.

## 1. Introduction

PBX1, which stands for pre-B-cell leukemia homeobox transcription factor 1, is an important transcription factor that plays a role in many physiological and developmental processes in humans [1]. It was first designated as *Prl*, an abbreviation for “pre-B-cell leukemia” [2,3]. Later, to prevent confusion with the prolactin gene *PRL*, it was renamed *PBX1* [4].The *PBX1* gene is located at chromosome 1q23.3, spanning more than 340 kb [1]. PBX1 is a member of the TALE (Three Aminoacid Loop Extension) family, a class of homeobox transcription factors [5,6]. The TALE class consists of two families, PBC (that includes PBX1-4) and MEINOX (that includes MEIS and PREP proteins) [5,6]. The PBX1 protein consists of 430 amino acids and contains 2 domains, PBC-A and PBC-B, that are the sites through which PBX1 binds to other proteins to carry out its function, and a homeodomain, which mediates DNA binding, and interaction with other proteins [5,6].

The PBX1 protein plays a crucial role in the regulation of gene expression during embryonic development and cell differentiation [1]. It is also essential for maintaining the pluripotency and self-renewal of stem cells and thereby it influences cell fate decisions by direct regulation of the expression of the key transcription factor NANOG [5,7]. A summary on PBX1’s involvement in the regulation of the hematopoietic and immune system is presented in Table 1.

Recent studies indicate that PBX1 is integral to the progression of various cancers [1,8]. Two separate research groups found that the homeobox domain of the gene is part of the chromosomal translocation t(1;19) in pre-B-cell acute lymphoblastic leukemia (ALL) [2,3]. PBX1 and several of its transcriptional targets have been associated with the regulation of signaling pathways involved in cell growth, invasion, metastasis, angiogenesis, resistance to cell death, and alterations in cellular energy utilization [1]. Furthermore, PBX1 may influence tumor-promoting inflammation and immune evasion, although the evidence supporting these claims is less established [1].

Understanding PBX1’s function and role in oncogenesis could aid researchers in identifying biomarkers that could enhance cancer prediction and diagnosis, as well as new therapeutic targets and strategies [8].

This review summarizes the impact of PBX1 on the development of hematologic malignancies, its influence on prognosis, and its potential use as a therapeutic target.

## 2. The Function of PBX1 in the Hematopoietic System

*PBX1* is a proto-oncogene and developmental regulator that maintains the balance between the self-renewal and differentiation of hematopoietic stem cells (HSCs) [9]. It can modulate the growth and differentiation of myeloid progenitors, either accelerating or decelerating these processes temporarily to safeguard its pool [9].

PBX1 is specifically involved in preserving hematopoietic stem cell potential for lymphoid, erythroid, and platelet lineages, to the detriment of other myeloid cell types [10]. PBX1 expression is essential for the differentiation of B-, T-, and NK cells, as well as megakaryocytes, and its deficiency mostly hinders B-cell maturation and megakaryocyte production [11].

PBX1 affects multiple genes associated with the commitment and maturation of B-cells and megakaryocytes, including *EBF1, PAX5*, *GATA1*, and *FOG1* and through complex transcriptional networks ensures their proper formation and function [10,12,13]. Moreover, through its interaction with *MN1* regulates HSC maintenance and self-renewal by participating in transcriptional regulation of target genes [8]. It interacts with genes such as *SMAD* and *Notch*, which are essential for T-cell differentiation and maturation [8,14,15,16]. It also regulates the expression of other relevant genes that influence the fate of thymic hematopoietic cells [14,15,16]. It functions as a transcriptional activator of NFIL3, facilitating the formation of natural killer cells [11].

In summary, PBX1 is a multifunctional protein that plays a role in hematopoietic stem cell self-renewal and the development of megakaryocytes, erythrocytes, and B- and T-lymphocytes [10].

## 3. The Role of PBX1 in Hematologic Neoplasms

The dysregulation of PBX1 has been linked to the initiation and advancement of multiple cancer types via the promotion of tumor cell proliferation through interactions with specific proteins or alterations in the transcription of target genes [1,5,8]. Its significance has been acknowledged in solid tumors, including breast, ovarian, renal, esophageal, and gastric carcinomas. It has also been examined in many hematological neoplasms, specifically multiple myeloma, Hodgkin lymphoma, myeloproliferative neoplasms (MPNs), and B-acute lymphoblastic leukemia (B-ALL) [5,6,17,18,19,20,21,22,23].

PBX1 overexpression occurs for several reasons, such as chromosomal translocations, like the t(1;19) translocation in pre-B-cell leukemia, which creates an oncogenic fusion protein. Aberrant signaling pathways, including those associated with estrogen receptors in breast cancer, can enhance PBX1 expression. Additional factors encompass mutations in regulatory elements or epigenetic alterations that augment PBX1 transcription, leading to its overexpression [6,8,24].

In the context of hematological malignancies, researchers have investigated its functional importance by employing mouse models that either lack PBX1 or have an excess of it [5,20,25,26]. At least ten hematological neoplasms have been linked to its aberrant expression. A summary on PBX1 interactions, which include gene upregulation, transcriptional regulatory complexes and signaling pathways in hematologic malignancies mainly found in mice and cell line experiments is presented in Table 2.

## 4. PBX1 in Leukemias

The translocation t(1;19) (q23; p13.3), is present in 3–5% of individuals with B-ALL and results in the creation of the E2A::PBX1 chimeric transcription factor [6]. This initial event provides the potential for self-renewal and leads to a susceptibility to further mutations in oncogenes that develop in B-ALL [5,6]. This fusion protein obstructs normal hematopoietic development, leading to the onset of ALL. The genes *BMI1*, an oncogene associated with lymphoid tissues, and *SETDB2*, a constituent of the protein lysine methyltransferase (PKMT) family that furthermore promotes the PI3K/AKT/mTOR signaling pathway, are prevalent in pre-B-cell ALL with E2A::PBX1 translocation [6,33,34,35]. In combination, these changes cause pre-B-cells to multiply. Furthermore, E2A::PBX1-mediated gene activation and leukemogenesis depend on how the fusion protein interacts with the MED1 subunit of the Mediator complex and on the coactivation of RUNX1 through direct connection to the PBX1 homeodomain [5,17,36]. This interaction renders the MED1 subunit a potential treatment objective in B-ALL with E2A::PBX1 translocation [5,17,36].

Preclinical evidence indicates that E2A::PBX1 contributes to the proliferation of T-acute lymphoblastic leukemia (T-ALL) in murine models. E2A::PBX1 appears to induce accelerated pro-T-cell proliferation, resulting in T-ALL. Since E2A::PBX1 does not attach to DNA, the process appears to rely on stem cell factors [8,37]. The interaction between E2A::PBX1 and HOX appears to facilitate the oncogenic transformation of T cells and the initiation of T-ALL in murine models [38]. Nevertheless, further investigation is necessary to comprehensively elucidate the essential connection and molecular mechanism [8,37,38].

In mixed-lineage leukemia (MLL)-rearranged ALL, the up-regulation of MEIS1 is crucial for sustaining the proliferation of leukemic cells; this homeobox transcription factor predominantly interacts with PBX1. Downregulation of MEIS1 may alter cellular responses to chemotherapy-induced apoptosis [6,39,40].

Researchers have examined the role of *HOX* genes (*HOXA9* and *HOXB3*) and *MEIS1* in the pathogenesis of acute myeloid leukemia (AML) in murine models. The MEIS1-PBX1 heterodimer seemingly binds to DNA, and their interaction promotes the development of AML by accelerating the leukemic transformation [27,28]. PREP1 is a homeobox protein belonging to the MEIS family that interacts with DNA by forming a heterodimer with PBX1. MEIS1-PBX1 and PREP1-PBX1 dimers can bind to DNA and form trimeric DNA-binding complexes with HOX proteins, which are essential for leukemic proliferation [27,28].

The oncogenic potential of the E2A::PBX1 fusion protein has been investigated in mice, demonstrating its involvement in the progression of AML by promoting the transformation of hematopoietic progenitor cells [41]. The oncogenic activity suggests that E2A::PBX1 interferes with normal differentiation and regulatory mechanisms in myeloid progenitors, facilitating leukemic transformation [41].

Overall, PBX1 promotes leukemogenesis by interacting with many genes, resulting in the dysregulation of self-renewal, proliferation, differentiation, and gene expression programs through epigenetic mechanisms.

## 5. PBX1 in Myeloproliferative Neoplasms and Myelodysplastic Syndromes

As previously stated, PBX1 is a crucial protein that regulates the equilibrium between self-renewal and differentiation in HSCs. It also influences myeloid progenitors, allowing them to sustain their population [10,23]. In certain MPN patients with the JAK2V617F mutation, an alteration in the copy number of *PBX1* was seen [42], accompanied by elevated expression levels in CD34+ cells in Polycythemia Vera (PV) patients [43]. Its involvement in JAK2 signaling was shown in mouse JAK2-deficient HSCs [23,44], and its overexpression in JAK-mutated HSCs appears to contribute to the MPN phenotype in vivo [23,45].

Research on the disease in murine models demonstrated the significant function of PBX1 in JAK2V617F-mutant MPN. In the absence of PBX1, the progression of MPN in mice was attenuated, despite the persistence of JAK2 mutation [23]. Thrombocytosis and leukocytosis were absent in the absence of PBX1, although initial erythrocytosis progressively diminished, and no other MPN-related symptoms were observed [23]. Furthermore, RNA sequencing results validated that PBX1 presence modulates various metabolic pathways affected by the JAK2V617F mutation in HPSCs [23]. This study demonstrates that PBX1 is essential for the maintenance of the malignant clone rather than the initiation of the disease [23].

PBX1 appears to be a significant factor in chronic myeloid leukemia (CML) as it promotes tumor cell proliferation through its interaction with *RNF6*, a direct target of PBX1 [6,29]. The HNRPDL/PBX1 axis also regulates the proliferation and imatinib sensitivity of CD34+ CML cells [6,29]. Elevated PBX1 levels inhibit cell proliferation through the silencing of HNRPDL [6,29].

Notwithstanding the previously cited data indicating PBX1’s involvement in MPNs, no studies have delineated its role in myelodysplastic syndromes (MDSs). Expression studies indicate that MDSs characterized by increased frequencies of long-term hematopoietic stem cells and multipotent progenitors generally express PBX1 [46].

## 6. PBX1 in Lymphomas

There is a paucity of data regarding the role of PBX1 in the development of lymphomas. However, the principal finding of PBX1 overexpression in Hodgkin lymphoma (HL) was noted in cell lines, especially in SUP-HD1 cells, a neoplastic Hodgkin-type cell line that secretes IFN-γ [5,22,47]. This overexpression is generally induced by duplication [5]. Biopsy tissue samples from patients with active disease have also been investigated, albeit to a lesser extent [22]. *PBX1* stimulates *NFIB*, an oncogene linked to several neoplasms, in HL cell lines, as demonstrated by elevated *NFIB* expression levels in the presence of PBX1 and diminished expression following PBX1 knockdown, thereby demonstrating PBX1’s direct regulatory effect on NFIB transcription [22,30]. The upregulation of all four members of the NFI family (*NFIA*, *NFIB*, *NFIC*, and *NFIX*) in patients with HL suggests their potential influence on disease progression [22]. Further research indicated that *TLX2*, a member of the NKL homeobox gene family, is an additional target gene of PBX1 in individuals with HL [22]. *TLX2* expression in HL cell lines is activated by *PBX1*, as evidenced by the correlation observed when PBX1 is present and the diminished expression following PBX1 knockdown [22]. The homeobox gene *TLX2* belongs to the transcription factor superfamily and is implicated in neural crest differentiation [48]. *PBX1* expression is inhibited in progenitor cells of the lymphocytic lineage and is downregulated during B-cell maturation. Thus, the continued presence of PBX1 positivity in mature B-cells may promote lymphoma formation [22].

The dysregulation of *PBX1*, independently or in combination with the *HOXB9* gene, leads to aberrant expression that disturbs B-cell maturation, thereby facilitating malignancy development [22,49]. Modifications of the *PBX1* gene have been verified in non-Hodgkin B-cell lymphomas, particularly Burkitt lymphoma and diffuse large B-cell lymphoma (DLBCL). A significant number of instances demonstrate *PBX1* fusion with *TCF3* [50]. This oncogenic translocation results in aberrant WNT signaling and an increased incidence of hematological neoplasia, impacting EBF3 and the WNT family members RORB and WNT16B [50]. In contrast, the role of *PBX1* in the advancement of DLBCL remains predominantly unexamined. A limited group of individuals with DLBCL and bone marrow involvement exhibited the 1q23-q25 translocation and the associated *PBX1* genetic mutation [51]. Regarding nodal disease, we are aware of only one documented case of a patient diagnosed with DLBCL and Langerhans cell histiocytosis of the inguinal lymph node [52]. Genetic analyses indicated that the patient exhibited a tetrasomy of *PBX1*. This genetic abnormality was probably linked to a poor outcome [52]. Finally, research utilizing murine models demonstrated that the E2A::PBX1 translocation is crucial to the pathogenesis of T-cell lymphoma, which differs from T-cell leukemia [8].

## 7. PBX1 in Multiple Myeloma

Multiple myeloma (MM) is a disease characterized by genetic and clinical heterogeneity [53]. Molecular abnormalities, primarily detected using FISH, are present in the majority of MM patients and are highly suggestive of prognosis [54]. Multiple myeloma often displays the t(4;14) translocation, a chromosomal anomaly linked to an unfavorable prognosis [54]. Previous studies have underscored the pivotal involvement of *PBX1* in the pathogenesis of MM, especially in cases involving the t(4;14) translocation [54,55]. In particular, the t(4;14) is associated with the overexpression of *PBX1*, which in complex with *MEIS2* is suggested to be involved in transcriptional regulation mediated by the *KLF4* gene, which is specifically overexpressed in t(4;14) patients. [54,55]. This implies that *PBX1* is involved in the transcriptional circuits that are linked to this particular molecular lesion in MM [6,54,55].

The genetic amplification of chromosome 1q21, the most prevalent copy number variation in MM, is well recognized as being related with a high disease burden, treatment resistance and dismal prognosis [20]. In addition to the various chr1q21 genes associated with a poor prognosis, other genes located on chr1q have recently been linked to the biology and prognosis of MM [20]. This discovery led to the hypothesis that the impact of chromosome 1q amplification in myeloma could be influenced by different regions along chromosome 1 [20]. A large-scale integrative analysis of clinical and multi-omics datasets revealed novel genes in the chr1q22 and chr1q23.3 regions that predict adverse prognosis for MM patients [20]. For instance, in myeloma cells displaying amplification of chromosome 1, ectopic expression of *PBX1*, situated at 1q23.3, has been documented [20]. The process involved genetic amplification and epigenetic activation across the entire domain [20]. The PBX1-FOXM1 axis controls critical oncogenic pathways or a transcriptional program that requires FOXM1 to promote cell proliferation [20]. PBX1 increases FOXM1 expression and its target genes, causing treatment resistance and poor prognosis [20]. The decrease in cell viability and cell cycle arrest after this axis disruption shows that it promotes the proliferative phenotype in MM and 1q amplification [20].

Recent research on a single-cell multiomics dataset from MM patients at different stages found proliferative states associated with high-risk cytogenetic events [31]. PHF19, an epigenetic regulator involved in gene expression control and chromatin remodeling, is particularly regulated by PBX1 [31]. *PHF19* expression in myeloma cell lines is greatly reduced by *PBX1* silencing, although *PBX1* amplification on 1q greatly enhances PHF19 levels [31]. The positive regulation of PHF19 by PBX1 affects cell proliferation and contributes to MM development [31]. High PHF19 levels are linked to aggressive disease characteristics such as enhanced proliferation and treatment resistance [31]. Thus, PBX1 and PHF19 may identify high-risk myeloma subtypes, necessitating more intensive treatment [20,31].

Recent studies have focused on the immunological evasion of MM cells from natural killer (NK) cells, specifically exosome-mediated lncRNA NEAT1 [32]. IncRNAs are RNA molecules that engage in biological processes but cannot transcribe into proteins [32]. Multiple myeloma exhibits lncRNA NEAT1 overexpression [32]. In a restricted study, exosomal NEAT1 by downregulating *PBX1* via the EZH2/PBX1 pathway, decreases NK-cell activity and lets MM cells avoid immunological detection [32].

## 8. Clinical Implications and Therapeutic Challenges

*PBX1* acts as a major regulator in cancer by orchestrating many oncogenic signaling pathways that facilitate tumor formation and progression [1,6]. In neoplasms, it plays a pivotal role in several processes, including angiogenesis, metastasis, apoptosis, and cellular proliferation and survival [1]. Moreover, PBX1 modulates gene expression and enhances chemoresistance and malignant stem cell characteristics by interacting with other transcription factors [1,6].

Several hematologic malignancies are linked to PBX1. It has been predominantly examined in ALL, a disorder that significantly contributes to leukemogenesis through the formation of the E2A::PBX1 fusion protein [5,6,8]. This protein arises from chromosomal translocation and acts as a powerful oncogene, facilitating the proliferation and survival of leukemic cells [5,6,8]. Information concerning the role of *PBX1* in adult lymphomas remains limited, requiring further research [5,21,22]. *PBX1* in MM patients with 1q amplification is a subject of considerable interest [19,20]. The overexpression of *PBX1* induces FOXM1 upregulation, thereby influencing the biologic behavior of the disease and the proliferation of myeloma cells [19,20]. The PBX1 level may also function as a biomarker in many malignancies. Nonetheless, further standardization and validation of the optimal measuring method are necessary for its application in clinical practice [1,5,6,8].

PBX1 could serve as a therapeutic target due to its interaction with various genes and pathways that facilitate cancer development [6]. This can be accomplished by modifying its activity or focusing on the unique PBX1-mediated pathway [6]. Numerous efforts have been undertaken to target PBX1 in a preclinical context, yielding promising outcomes thus far [6]. The therapeutic strategy of targeting HOX/PBX dimers was advantageous in AML [56]. Cases of B-ALL exhibiting translocation t(1;19) can be also treated with agents that diminish E2A::PBX1 expression [57]. Additionally, drugs that interfere with the PBX1-FOXM1 axis or PBX1 small-molecule inhibitors have been proposed as potential treatment alternatives for chr1q-amp myeloma [19]. T417 is a small molecule that inhibits the transcriptional activity of PBX1 [58]. The compound effectively interferes with PBX1-DNA interactions and thereby destabilizing them and thus reducing the expression of PBX1 target genes [58]. Its potential benefit has been previously reported in other types of cancers [58] and most recently, its selective activity against chr1q21 amplified MM cells has been reported in a preclinical setting [20]. To fully understand the therapeutic potential of PBX1 interactions, further research and clinical studies are necessary [6,8].

## 9. Conclusions

In conclusion, PBX1 and its variant E2A::PBX1 function as transcription factors, engaging with several proteins, including HOX, MEIS, and PREP, to modulate gene expression. PBX1 is crucial for the regulation of cell proliferation and differentiation in hematopoietic lineages. The complexity of PBX1 makes it a compelling subject for further research to thoroughly elucidate the specific mechanisms by which this molecule governs cellular processes, its influence on the development of both mature and immature hematologic neoplasms, and its viability as a biomarker. Furthermore, to translate initial findings into viable antineoplastic treatments, further clinical studies of PBX1-targeted medicines are necessary. We assert that the development of targeted therapeutics for malignancies associated with PBX1 and E2A::PBX1 will gain significance as research on their oncogenic mechanisms advances.

## Figures and Tables

**Table 1:** PBX1’s involvement in the regulation of hematopoietic and immune systems [8].

System	Signaling Pathway	Function
Hematopoietic system	Regulation of *EF1*, *PAX5*, *GATA1*, *FOG1*, *MN1*	Supports HSC self-renewal and differentiation
		B-cell and megakaryocyte expansion and maturation
Immune system	Upregulation of the AKT1 pathway via activation by ILT2 receptor Direct activation of Rtkn2 expression Direct regulation of CD44 expression	Maturation and function of several immune cells (NK cells, T reg cells, T cells)
Thymus	Regulates PAX1 expression Interactions with *SMAD*, *Notch*	Regulates the proliferation of thymocytes
Spleen	Upregulation of *Nkx2-5* Downregulation of *p15Ink4b*	Splenic cell development and function

**Table 2:** A summary of the main PBX1 interactions in hematologic neoplasms.

Hematologic Neoplasm	Gene Upregulation, Transcriptional Regulators and Signaling Pathways	Function
Acute lymphoblastic leukemia	E2A::PBX1	Impedes normal hematopoiesis [6]
Acute myeloid leukemia	MEIS1-PBX1 interaction (transcriptional regulatory complex)	Binds to DNA and facilitates leukemic transformation [27,28]
	PREP-PBX1 interaction	Binds to DNA and creates complexes with HOX proteins inducing leukemic cell proliferation [27,28]
Mixed-lineage leukemia	*MEIS1* upregulation	Primarily interacts with PBX1 and promotes proliferation of leukemic cells [6]
Philadelphia-negative MPN	*PBX1* upregulation	Sustain malignant clone of MPN [23]
Chronic myeloid leukemia	HNRPDL/PBX1 axis	Controls growth and imatinib sensitivity of CD34+ CML cells [6,29]
Hodgkin lymphoma	NFIB upregulation	Activated by *PBX1* [22,30]
	TLX2 upregulation	Activated by *PBX1* [22,30]
Multiple myeloma	PBX1-FOXM1 signaling axis	Drives proliferative phenotype in 1q amp MM [20]
	*PHF19* upregulation	PBX1 is a positive regulator of *PHF19*, which affects cell proliferation in MM [31]
	lncRNA NEAT1 overexpression	Inhibits NK-cell activity and therefore promotes immune escape [32]

## Data Availability

Not applicable.

## Abbreviations

The following abbreviations are used in this manuscript:

ALL	Acute lymphoblastic leukemia
B-ALL	B-Acute lymphoblastic leukemia
CML	Chronic myeloid leukemia
DLBCL	Diffuse large B-cell lymphoma
EBF	Early B-cell factor
FOG1	Forkhead box G1
FOXM1	Forkhead box protein M1
HL	Hodgkin lymphoma
HNRPDL	Heterogeneous nuclear ribonucleoprotein D-like
HOX	Homeobox
HSCs	Hematopoietic stem cells
lncRNA	Long noncoding RNAs
MDS	Myelodysplastic syndromes
MEIS1	Myeloid ecotropic viral integration site 1
MLL	Mixed lineage leukemia
MM	Multiple myeloma
MPN	Myeloproliferative neoplasm
NFI	Nuclear factor I
NFIL3	Nuclear factor interleukin 3 regulated
NK	Natural Killer
PAX5	Paired box 5
PBX1	Pre-B-cell leukemia factor 1
PHF19	PHD finger protein 19
PKMT	Protein lysine methyltransferase
PREP1	PBX-regulating protein 1
PV	Polycythemia vera
RNF6	Ring finger protein 6
RORB	RAR-related orphan receptor beta
RT-qPCR	Real time quantitative polymerase chain reaction
TALE	Three amino acid loop extension
T-ALL	T-Acute lymphoblastic leukemia
TLX2	T-cell leukemia homeobox 2

## References

[1] Veiga R.N., de Oliveira J.C., Gradia D.F. (2021). PBX1: A Key Character of the Hallmarks of Cancer. J. Mol. Med..

[2] Kamps M.P., Murre C., Sun X.H., Baltimore D. (1990). A New Homeobox Gene Contributes the DNA Binding Domain of the t(1;19) Translocation Protein in Pre-B ALL. Cell.

[3] Nourse J., Mellentin J.D., Galili N., Wilkinson J., Stanbridge E., Smith S.D., Cleary M.L. (1990). Chromosomal Translocation t(1;19) Results in Synthesis of a Homeobox Fusion mRNA That Codes for a Potential Chimeric Transcription Factor. Cell.

[4] Kamps M.P., Look A.T., Baltimore D. (1991). The Human t(1;19) Translocation in Pre-B ALL Produces Multiple Nuclear E2A-Pbx1 Fusion Proteins with Differing Transforming Potentials. Genes Dev..

[5] Crisafulli L., Brindisi M., Liturri M.G., Sobacchi C., Ficara F. (2024). PBX1: A TALE of Two Seasons-Key Roles during Development and in Cancer. Front. Cell Dev. Biol..

[6] Kao T.-W., Chen H.-H., Lin J., Wang T.-L., Shen Y.-A. (2024). PBX1 as a Novel Master Regulator in Cancer: Its Regulation, Molecular Biology, and Therapeutic Applications. Biochim. Biophys. Acta Rev. Cancer.

[7] Chan K.K.-K., Zhang J., Chia N.-Y., Chan Y.-S., Sim H.S., Tan K.S., Oh S.K.-W., Ng H.-H., Choo A.B.-H. (2009). KLF4 and PBX1 Directly Regulate NANOG Expression in Human Embryonic Stem Cells. Stem Cells.

[8] Liu M., Xing Y., Tan J., Chen X., Xue Y., Qu L., Ma J., Jin X. (2024). Comprehensive Summary: The Role of PBX1 in Development and Cancers. Front. Cell Dev. Biol..

[9] Ficara F., Murphy M.J., Lin M., Cleary M.L. (2008). Pbx1 Regulates Self-Renewal of Long-Term Hematopoietic Stem Cells by Maintaining Their Quiescence. Cell Stem Cell..

[10] Ficara F., Crisafulli L., Lin C., Iwasaki M., Smith K.S., Zammataro L., Cleary M.L. (2013). Pbx1 Restrains Myeloid Maturation While Preserving Lymphoid Potential in Hematopoietic Progenitors. J. Cell Sci..

[11] Xu X., Zhou Y., Fu B., Zhang J., Dong Z., Zhang X., Shen N., Sun R., Tian Z., Wei H. (2020). PBX1 Promotes Development of Natural Killer Cells by Binding Directly to the Nfil3 Promoter. FASEB J..

[12] Cullmann K., Jahn M., Spindler M., Schenk F., Manukjan G., Mucci A., Steinemann D., Boller K., Schulze H., Bender M. (2020). Forming Megakaryocytes from Murine Induced Pluripotent Stem Cells by the Inducible Overexpression of Supporting Factors. Res. Pract. Thromb. Haemost..

[13] Zewdu R., Risolino M., Barbulescu A., Ramalingam P., Butler J.M., Selleri L. (2016). Spleen Hypoplasia Leads to Abnormal Stress Hematopoiesis in Mice with Loss of Pbx Homeoproteins in Splenic Mesenchyme. J. Anat..

[14] Jurberg A.D., Vasconcelos-Fontes L., Cotta-de-Almeida V. (2015). A Tale from TGF-β Superfamily for Thymus Ontogeny and Function. Front. Immunol..

[15] McCarron M.J., Irla M., Sergé A., Soudja S.M., Marie J.C. (2019). Transforming Growth Factor-Beta Signaling in Aβ Thymocytes Promotes Negative Selection. Nat. Commun..

[16] Mary L., Leclerc D., Labalme A., Bellaud P., Mazaud-Guittot S., Dréano S., Evrard B., Bigand A., Cauchoix A., Loget P. (2023). Functional Assessment of a New PBX1 Variant in a 46,XY Fetus with Severe Syndromic Difference of Sexual Development through CRISPR-Cas9 Gene Editing. Genes.

[17] Pi W.-C., Wang J., Shimada M., Lin J.-W., Geng H., Lee Y.-L., Lu R., Li D., Wang G.G., Roeder R.G. (2020). E2A-PBX1 Functions as a Coactivator for RUNX1 in Acute Lymphoblastic Leukemia. Blood.

[18] Yin H., Wang J., Tan Y., Jiang M., Zhang H., Meng G. (2023). Transcription Factor Abnormalities in B-ALL Leukemogenesis and Treatment. Trends Cancer.

[19] Trasanidis N., Katsarou A., Bergonia B., Keren K., Kostopoulos I.V., Ponnusamy K., Paudel R., Xiao X., Auner H.W., Roberts I. (2019). PBX1 Co-Operates with FOXM1 to Regulate Myeloma Cell Proliferation and to Define an Ultra High-Risk chr1q Gain Myeloma Patient Subgroup. Blood.

[20] Trasanidis N., Katsarou A., Ponnusamy K., Shen Y.-A., Kostopoulos I.V., Bergonia B., Keren K., Reema P., Xiao X., Szydlo R.M. (2022). Systems Medicine Dissection of Chr1q-Amp Reveals a Novel PBX1-FOXM1 Axis for Targeted Therapy in Multiple Myeloma. Blood.

[21] Nagel S., Meyer C., Pommerenke C. (2023). Establishment of the Lymphoid ETS-Code Reveals Deregulated ETS Genes in Hodgkin Lymphoma. PLoS ONE.

[22] Nagel S., Pommerenke C., Meyer C., MacLeod R.A.F., Drexler H.G. (2021). Establishment of the TALE-Code Reveals Aberrantly Activated Homeobox Gene PBX1 in Hodgkin Lymphoma. PLoS ONE.

[23] Muggeo S., Crisafulli L., Uva P., Fontana E., Ubezio M., Morenghi E., Colombo F.S., Rigoni R., Peano C., Vezzoni P. (2021). PBX1-Directed Stem Cell Transcriptional Program Drives Tumor Progression in Myeloproliferative Neoplasm. Stem Cell Rep..

[24] Panda T., Rainchwar S., Singh R., Singh A., Soni M., Kakkar D., Jegan K.R., Pillai R.H., Palatty R.J., Jha K. (2024). Real World Outcome of B ALL with t (1; 19) (Q23; P13)/TCF3::PBX1 in Adolescents and Adults Treated with Intensive Regimes. Leuk. Res..

[25] Park J.T., Shih I.-M., Wang T.-L. (2008). Identification of Pbx1, a Potential Oncogene, as a Notch3 Target Gene in Ovarian Cancer. Cancer Res..

[26] Thiaville M.M., Stoeck A., Chen L., Wu R.-C., Magnani L., Oidtman J., Shih I.-M., Lupien M., Wang T.-L. (2012). Identification of PBX1 Target Genes in Cancer Cells by Global Mapping of PBX1 Binding Sites. PLoS ONE.

[27] Moskow J.J., Bullrich F., Huebner K., Daar I.O., Buchberg A.M. (1995). Meis1, a PBX1-Related Homeobox Gene Involved in Myeloid Leukemia in BXH-2 Mice. Mol. Cell Biol..

[28] Thorsteinsdottir U., Kroon E., Jerome L., Blasi F., Sauvageau G. (2001). Defining Roles for HOX and MEIS1 Genes in Induction of Acute Myeloid Leukemia. Mol. Cell Biol..

[29] Ji D., Zhang P., Ma W., Fei Y., Xue W., Wang Y., Zhang X., Zhou H., Zhao Y. (2020). Oncogenic Heterogeneous Nuclear Ribonucleoprotein D-like Modulates the Growth and Imatinib Response of Human Chronic Myeloid Leukemia CD34+ Cells via Pre-B-Cell Leukemia Homeobox 1. Oncogene.

[30] Chen K.-S., Lim J.W.C., Richards L.J., Bunt J. (2017). The Convergent Roles of the Nuclear Factor I Transcription Factors in Development and Cancer. Cancer Lett..

[31] Johnson T.S., Sudha P., Liu E., Becker N., Robertson S., Blaney P., Morgan G., Chopra V.S., Dos Santos C., Nixon M. (2024). 1q Amplification and PHF19 Expressing High-Risk Cells Are Associated with Relapsed/Refractory Multiple Myeloma. Nat. Commun..

[32] Wang Q.-M., Lian G.-Y., Sheng S.-M., Xu J., Ye L.-L., Min C., Guo S.-F. (2024). Exosomal lncRNA NEAT1 Inhibits NK-Cell Activity to Promote Multiple Myeloma Cell Immune Escape via an EZH2/PBX1 Axis. Mol. Cancer Res..

[33] Grüninger P.K., Uhl F., Herzog H., Gentile G., Andrade-Martinez M., Schmidt T., Han K., Morgens D.W., Bassik M.C., Cleary M.L. (2022). Functional Characterization of the PI3K/AKT/MTOR Signaling Pathway for Targeted Therapy in B-Precursor Acute Lymphoblastic Leukemia. Cancer Gene Ther..

[34] Smith K.S., Chanda S.K., Lingbeek M., Ross D.T., Botstein D., van Lohuizen M., Cleary M.L. (2003). Bmi-1 Regulation of INK4A-ARF Is a Downstream Requirement for Transformation of Hematopoietic Progenitors by E2a-Pbx1. Mol. Cell.

[35] Lin C.-H., Wong S.H.-K., Kurzer J.H., Schneidawind C., Wei M.C., Duque-Afonso J., Jeong J., Feng X., Cleary M.L. (2018). SETDB2 Links E2A-PBX1 to Cell-Cycle Dysregulation in Acute Leukemia through CDKN2C Repression. Cell Rep..

[36] Lee Y.-L., Ito K., Pi W.-C., Lin I.-H., Chu C.-S., Malik S., Cheng I.-H., Chen W.-Y., Roeder R.G. (2021). Mediator Subunit MED1 Is Required for E2A-PBX1-Mediated Oncogenic Transcription and Leukemic Cell Growth. Proc. Natl. Acad. Sci. USA.

[37] Sykes D.B., Kamps M.P. (2004). E2a/Pbx1 Induces the Rapid Proliferation of Stem Cell Factor-Dependent Murine pro-T Cells That Cause Acute T-Lymphoid or Myeloid Leukemias in Mice. Mol. Cell Biol..

[38] Bijl J., Krosl J., Lebert-Ghali C.-E., Vacher J., Mayotte N., Sauvageau G. (2008). Evidence for Hox and E2A-PBX1 Collaboration in Mouse T-Cell Leukemia. Oncogene.

[39] Rosales-Aviña J.A., Torres-Flores J., Aguilar-Lemarroy A., Gurrola-Díaz C., Hernández-Flores G., Ortiz-Lazareno P.C., Lerma-Díaz J.M., de Celis R., González-Ramella Ó., Barrera-Chaires E. (2011). MEIS1, PREP1, and PBX4 Are Differentially Expressed in Acute Lymphoblastic Leukemia: Association of MEIS1 Expression with Higher Proliferation and Chemotherapy Resistance. J. Exp. Clin. Cancer Res..

[40] Blasi F., Bruckmann C. (2021). MEIS1 in Hematopoiesis and Cancer. How MEIS1-PBX Interaction Can Be Used in Therapy. J. Dev. Biol..

[41] Kamps M.P., Baltimore D. (1993). E2A-Pbx1, the t(1;19) Translocation Protein of Human Pre-B-Cell Acute Lymphocytic Leukemia, Causes Acute Myeloid Leukemia in Mice. Mol. Cell Biol..

[42] Rice K.L., Lin X., Wolniak K., Ebert B.L., Berkofsky-Fessler W., Buzzai M., Sun Y., Xi C., Elkin P., Levine R. (2011). Analysis of Genomic Aberrations and Gene Expression Profiling Identifies Novel Lesions and Pathways in Myeloproliferative Neoplasms. Blood Cancer J..

[43] Berkofsky-Fessler W., Buzzai M., Kim M.K.-H., Fruchtman S., Najfeld V., Min D.-J., Costa F.F., Bischof J.M., Soares M.B., McConnell M.J. (2010). Transcriptional Profiling of Polycythemia Vera Identifies Gene Expression Patterns Both Dependent and Independent from the Action of JAK2V617F. Clin. Cancer Res..

[44] Akada H., Akada S., Hutchison R.E., Sakamoto K., Wagner K.-U., Mohi G. (2014). Critical Role of Jak2 in the Maintenance and Function of Adult Hematopoietic Stem Cells. Stem Cells.

[45] Shepherd M.S., Li J., Wilson N.K., Oedekoven C.A., Li J., Belmonte M., Fink J., Prick J.C.M., Pask D.C., Hamilton T.L. (2018). Single-Cell Approaches Identify the Molecular Network Driving Malignant Hematopoietic Stem Cell Self-Renewal. Blood.

[46] Ganan-Gomez I., Yang H., Ma F., Montalban-Bravo G., Thongon N., Marchica V., Richard-Carpentier G., Chien K., Manyam G., Wang F. (2022). Stem Cell Architecture Drives Myelodysplastic Syndrome Progression and Predicts Response to Venetoclax-Based Therapy. Nat. Med..

[47] Naumovski L., Utz P.J., Bergstrom S.K., Morgan R., Molina A., Toole J.J., Glader B.E., McFall P., Weiss L.M., Warnke R. (1989). SUP-HD1: A New Hodgkin’s Disease-Derived Cell Line with Lymphoid Features Produces Interferon-Gamma. Blood.

[48] Borghini S., Bachetti T., Fava M., Di Duca M., Cargnin F., Fornasari D., Ravazzolo R., Ceccherini I. (2006). The TLX2 Homeobox Gene Is a Transcriptional Target of PHOX2B in Neural-Crest-Derived Cells. Biochem. J..

[49] Nagel S. (2022). The NKL- and TALE-Codes Represent Hematopoietic Gene Signatures to Evaluate Deregulated Homeobox Genes in Hodgkin Lymphoma. Hemato.

[50] OncoKB^TM^—MSK’s Precision Oncology Knowledge Base. https://www.oncokb.org/.

[51] Kim S., Kim H., Kang H., Kim J., Eom H., Kim T., Yoon S.-S., Suh C., Lee D., Korean Society of Hematology Lymphoma Working Party (2013). Clinical Significance of Cytogenetic Aberrations in Bone Marrow of Patients with Diffuse Large B-Cell Lymphoma: Prognostic Significance and Relevance to Histologic Involvement. J. Hematol. Oncol..

[52] Moraveji S., Tonk V., Gaur S., Torabi A. (2016). Langerhans Cell Histiocytosis and Diffuse Large B-Cell Lymphoma with Tetrasomy of PBX1 Gene and t(14;19): Two Entities in One Lymph Node. Pathology.

[53] Kumar S.K., Rajkumar S.V. (2018). The Multiple Myelomas—Current Concepts in Cytogenetic Classification and Therapy. Nat. Rev. Clin. Oncol..

[54] Abdallah N., Rajkumar S.V., Greipp P., Kapoor P., Gertz M.A., Dispenzieri A., Baughn L.B., Lacy M.Q., Hayman S.R., Buadi F.K. (2020). Cytogenetic Abnormalities in Multiple Myeloma: Association with Disease Characteristics and Treatment Response. Blood Cancer J..

[55] Calura E., Bisognin A., Manzoni M., Todoerti K., Taiana E., Sales G., Morgan G.J., Tonon G., Amodio N., Tassone P. (2016). Disentangling the microRNA Regulatory Milieu in Multiple Myeloma: Integrative Genomics Analysis Outlines Mixed miRNA-TF Circuits and Pathway-Derived Networks Modulated in t(4;14) Patients. Oncotarget.

[56] Alharbi R.A., Pandha H.S., Simpson G.R., Pettengell R., Poterlowicz K., Thompson A., Harrington K., El-Tanani M., Morgan R. (2017). Inhibition of HOX/PBX Dimer Formation Leads to Necroptosis in Acute Myeloid Leukemia Cells. Oncotarget.

[57] Uckun F.M., Qazi S., Dibirdik I., Myers D.E. (2013). Rational Design of an Immunoconjugate for Selective Knock-down of Leukemia-Specific E2A-PBX1 Fusion Gene Expression in Human Pre-B Leukemia. Integr. Biol..

[58] Shen Y.-A., Jung J., Shimberg G.D., Hsu F.-C., Rahmanto Y.S., Gaillard S.L., Hong J., Bosch J., Shih I.-M., Chuang C.-M. (2021). Development of Small Molecule Inhibitors Targeting PBX1 Transcription Signaling as a Novel Cancer Therapeutic Strategy. iScience.

